# Exploring the antecedents of tourist satisfaction: A big data analysis of ice and snow tourism destinations

**DOI:** 10.1371/journal.pone.0358229

**Published:** 2026-09-15

**Authors:** Shuo Wang, Shiyang Chen, Qingyang Guan

**Affiliations:** Harbin University of Commerce, Harbin, Heilongjiang Province, China; Zhejiang Agriculture and Forestry University: Zhejiang A and F University, CHINA

## Abstract

This study explores tourist satisfaction in winter destinations using a big-data approach that integrates Latent Dirichlet Allocation, sentiment analysis, and Vector Autoregression. Drawing on over 32,000 online reviews from China’s ice and snow tourism sites, the research identifies key concerns—such as infrastructure, service quality, and pricing—and reveals strong seasonal and emotional variability. A central finding is the asymmetrical impact of emotion: negative sentiments, especially regarding perceived price unfairness, have a greater influence on satisfaction than positive emotions. The study contributes theoretically by demonstrating the dominance of emotional drivers in satisfaction formation and introducing a scalable, dynamic framework for modeling affective-cognitive interactions over time. These insights highlight the value of emotion-sensitive management strategies in cold-region tourism and offer a methodological foundation for future behavioral research in dynamic tourism contexts.

## 1. Introduction

Ice and snow tourism has become an increasingly important and dynamic component of the global tourism market, especially in high-latitude countries such as Sweden, Canada, Japan, and China Jiang [1]. In recent years, the growing interest in seasonal, experience-based travel has significantly boosted demand for unique winter tourism products, including skiing, snowboarding, and large-scale snow festivals. In response, many regions have invested heavily in winter sports infrastructure, creating competitive and attractive destinations for both domestic and international tourists. In China, ice and snow tourism is not only a leisure activity but also a strategic sector for regional economic development. A prominent example is the Harbin International Ice and Snow Sculpture Festival, which attracted more than 23 million visitors during the 2022–2023 winter season and generated over RMB 30.9 billion (approximately USD 4.5 billion) in tourism revenue. This festival illustrates how ice and snow tourism can serve as an important driver of local economic development and cultural visibility on the global stage. According to CGTN [2], the 2022–2023 winter season attracted over 23 million visitors and generated RMB 30.9 billion in tourism revenue.

Despite its rapid expansion and growing economic importance, ice and snow tourism continues to face several persistent challenges that may limit its long-term development potential. One of the most critical issues is seasonal variability, as many destinations experience short and unpredictable winters, which directly affect the reliability and attractiveness of tourism activities, as noted by François et al. and Richins [3,4]. The increasing dependence on artificial snowmaking in areas with shorter winters raises concerns about environmental costs and long-term sustainability. Furthermore, accurately capturing tourist satisfaction in this context remains difficult, as conventional survey methods may fail to reflect the complex emotional and cognitive aspects of the tourist experience.

With the development of big data technologies, researchers now have new tools to explore tourist satisfaction by analyzing online reviews. Methods such as Latent Dirichlet Allocation (LDA) for topic extraction and sentiment analysis tools like SnowNLP enable the meaningful interpretation of large volumes of unstructured textual data, as demonstrated by Huang and Chelliah and Guo et al. [5,6]. These methods facilitate the identification of key topics and sentiments expressed by tourists, providing a more comprehensive understanding of their experiences.

Building upon this foundation, the present study employs an integrated analytical framework combining LDA, sentiment analysis, and Vector Autoregression (VAR) modeling to investigate the sentiment experiences and satisfaction drivers of tourists in ice and snow destinations. Through analyzing ice and snow tourism, this research aims to address the following questions:

*(1) What are the emotional experiences of tourists at ice and snow tourism destinations?*
*(2) What are the key concerns of tourists when engaging in ice and snow tourism?*
*(3) How do emotional experiences influence tourist satisfaction in this context?*

This study offers several theoretical contributions to the literature on tourist satisfaction and ice and snow tourism. First, it advances satisfaction research by framing tourist satisfaction as a dynamic and evolving process rather than a static post-visit evaluation. By modeling sentiment and satisfaction over time, the study demonstrates that satisfaction is continuously shaped by emotional fluctuations, thereby extending traditional outcome-oriented perspectives toward a process-based understanding. Second, the findings deepen theoretical insights into emotion-driven satisfaction formation by showing that negative emotions exert a stronger and more persistent influence on satisfaction than positive emotions in ice and snow tourism contexts. This asymmetric effect highlights the heightened emotional sensitivity of tourists in highly seasonal and capacity-constrained environments and underscores the central role of affective mechanisms in shaping overall evaluations. Third, the study contributes to tourism theory by integrating cognitive themes and emotional responses within a unified analytical framework. By jointly examining what tourists experience and how they emotionally react, the research provides a more comprehensive explanation of satisfaction formation than approaches that focus on either experiential attributes or emotions alone. Finally, this research enriches the theoretical understanding of ice and snow tourism by contextualizing satisfaction mechanisms within a rapidly developing and policy-driven tourism system. The results illustrate how large-scale development, seasonal concentration, and digitally mediated experiences interact to shape tourist satisfaction in ways that differ from traditional winter tourism destinations. The remainder of this paper is organized as follows. Section 2 reviews the relevant literature on ice and snow tourism, tourist satisfaction, and big data analytics in tourism research. Section 3 introduces the study area, data sources, research framework, and methodological approaches, including LDA topic modeling, sentiment analysis, and VAR modeling. Section 4 presents the empirical results and discusses the thematic patterns, seasonal sentiment dynamics, and temporal causal relationships among satisfaction dimensions. Finally, Section 5 summarizes the main conclusions, theoretical contributions, managerial implications, and limitations for future research.

## 2. Literature review

This section reviews the existing literature related to ice and snow tourism, tourist satisfaction, and big data analytics in tourism research. It first examines the characteristics and development challenges of ice and snow tourism, followed by a discussion of tourist satisfaction theories and emotional experience studies. Subsequently, the section reviews the application of big data and sentiment analysis methods in tourism research and concludes by identifying the major research gaps addressed in the present study.

### 2.1. Ice and snow tourism

Ice and snow tourism refers to travel that centers around destinations with winter-related activities, such as skiing, snowboarding, snowshoeing, ice climbing, and various winter leisure pursuits. This type of tourism is especially popular in colder climates, where natural elements such as snowfall and low temperatures create unique opportunities for outdoor recreation. It is distinct from other forms of tourism due to its heavy reliance on environmental factors, primarily consistent snow cover and cold temperatures, which determine both the seasonality and attractiveness of the destinations. The viability of ice and snow tourism is largely tied to the reliability of climatic conditions, making it particularly vulnerable to the impacts of climate change. Research has demonstrated that warming temperatures and fluctuating snowfall patterns are diminishing the length and reliability of ski seasons in many regions, highlighting growing climate-related challenges for winter tourism destinations, as reported by Scott et al. [7]. Research has demonstrated that warming temperatures and fluctuating snowfall patterns are diminishing the length and reliability of ski seasons in many regions, highlighting growing climate-related challenges for winter tourism destinations, as reported by Steiger et al. [8]. This shift not only challenges tourist satisfaction but also poses significant economic risks for destinations that depend heavily on winter tourism. The potential economic consequences of these changes are particularly concerning for communities where tourism constitutes a major source of income, as emphasized by Timoshenko [9].

Adaptive measures such as the development of snowmaking technologies, the implementation of energy-efficient infrastructure, and the diversification of tourism products through year-round recreational activities have been proposed to mitigate the impacts of climate change. Such adaptation strategies can help maintain the attractiveness and competitiveness of tourism destinations under changing environmental conditions, as highlighted by Hao [10]. Similarly, integrating local cultural experiences and promoting off-season tourism activities can diversify tourism offerings and reduce dependence on snow reliability, as emphasized by Richards [11].

Furthermore, studies have explored the importance of destination resilience, emphasizing the need for strategic planning that incorporates both environmental sustainability and the diversification of tourism experiences. Diversified tourism products, including cultural tours, winter festivals, and eco-tourism initiatives, can enhance the resilience of ice and snow tourism destinations by reducing dependence on a single tourism resource. The incorporation of such offerings can not only strengthen destination attractiveness but also enrich tourists’ overall experiences, as argued by Dawson and Scott [12].

Although ice and snow tourism has been widely examined in mature winter tourism destinations such as Canada and Norway, existing findings are largely grounded in long-established recreational cultures and stable market structures. Research in these contexts has primarily focused on climate-related risks, destination adaptation, and the sustainability of winter tourism systems, as demonstrated by Scott et al. and Steiger et al. [7,8]. In these contexts, winter tourism has evolved gradually over decades, with repeat visitors, well-developed infrastructure, and relatively predictable demand patterns. As a result, prior studies often conceptualize tourist satisfaction primarily in terms of service quality, snow reliability, and destination image within relatively stable operating environments. In contrast, China’s ice and snow tourism has developed under markedly different institutional and market conditions. Its rapid expansion has been strongly influenced by national policy initiatives, including the post-Olympic development agenda and large-scale participation campaigns. These measures have accelerated destination development and tourism demand growth within a relatively short period, as highlighted by Jiang [1], Such policy-driven development can generate seasonal congestion, capacity mismatches, and heightened service pressure, potentially intensifying tourists’ emotional responses during peak periods. Moreover, visitor structure differs substantially. While winter tourism markets in Canada and Norway are characterized by high levels of repeat participation and skill-based recreation, China’s ice and snow tourism market includes a large proportion of first-time or infrequent participants, many of whom originate from regions without natural snow conditions. Research in mature winter tourism markets has primarily focused on climate-related risks, destination adaptation, and the sustainability of winter tourism systems, as demonstrated by Scott et al. [7]. This distinctive market structure contributes to unique patterns of destination perception and tourist behavior, as noted by Liu and Guo [13]. These tourists may rely more heavily on symbolic and experiential cues such as festival atmosphere, snow landscapes, and entertainment activities when forming satisfaction judgments. Consequently, emotional reactions to crowding, pricing, and service delivery may play a more prominent role in shaping overall evaluations. In addition, the experiential configuration of ice and snow tourism in China extends beyond sport-centered activities. Whereas research in traditional winter destinations focuses predominantly on skiing and snow-based recreation, Chinese ice and snow destinations frequently integrate large-scale festivals, themed architecture, and immersive entertainment environments. This hybrid experiential structure suggests that satisfaction formation may involve stronger affective and symbolic components, rather than being driven solely by functional performance attributes. Finally, the high penetration of online platforms in China has resulted in extensive user-generated content that records tourists’ emotional expressions in real time. This digitally mediated environment provides both a distinctive behavioral context and a rich empirical basis for analyzing how emotions dynamically shape satisfaction. Taken together, these contextual differences indicate that conclusions derived from mature Western winter tourism markets may not be directly transferable to China. A dedicated empirical investigation is therefore necessary to examine whether and how emotional dynamics influence tourist satisfaction within this rapidly developing and policy-driven ice and snow tourism system.

However, a key factor influencing the sustainability of ice and snow tourism lies in tourists’ satisfaction. Beyond snow conditions, satisfaction is shaped by service quality, infrastructure, and emotional experiences. Understanding these perceptions is essential for maintaining destination appeal in the face of climate uncertainties.

### 2.2. Tourists’ satisfaction about ice and snow tourism

Tourist satisfaction is a nuanced and multifaceted concept in ice and snow tourism, given its reliance not only on environmental conditions but also on a complex interplay among service quality, infrastructure, and emotional experience. Traditional research has predominantly employed structured questionnaires, interviews, and panel data to explore this phenomenon. Even relatively small variations in snow depth can significantly influence tourist satisfaction, as demonstrated through dynamic panel data analysis by Falk [14]. User-generated photographs have also been shown to serve as effective proxies for evaluating destination image and aligning tourists’ expectations, as demonstrated by Kim and Stepchenkova [15] Furthermore, positive emotional experiences characterized by joy, affection, and surprise are closely associated with tourist satisfaction and word-of-mouth intentions, based on the multidimensional emotional framework developed by Hosany et al. [16]. The importance of understanding the relationship between emotional responses and tourism experience evaluation has been further emphasized by Zhang [17]. However, these methods exhibit several noteworthy limitations: surveys and interviews are typically conducted post-experience and so lack the capacity to capture real-time fluctuations in tourist sentiment; the convenience-based sampling often produces low sample sizes that compromise representativeness; and psychometric scales, despite rigorous design, fail to uncover subtle or emergent emotions embedded in spontaneous online reviews. Indeed, textual analyses of ice and snow tourism reviews suggest that sentiment dynamics during tourism experiences often associated with factors such as pricing transparency, service quality, and on-site delays are considerably more complex and fluid than can be captured by traditional survey-based approaches. Tourist satisfaction and evaluation are influenced by multiple interrelated experiential factors that evolve dynamically throughout the travel process, as emphasized by Puh and Babac [18]. Moreover, while sentiment analysis models (e.g., IDCAN-BiLSTM) developed for this niche have achieved over 92% classification accuracy, they underscore the need for computational processing of large-scale textual data to decipher granular emotional patterns in near real-time. These shortcomings signal a clear methodological gap: conventional tools are ill-suited to track the temporal evolution and contextual nuances of tourist satisfaction in ice and snow environments. As such, there is a growing imperative to adopt more scalable, dynamic, and data-driven approaches to better understand tourist experiences in this specialized sector.

### 2.3. Big data analysis in ice and snow tourism

The emergence of big data analytics has profoundly transformed tourism research, providing novel insights into tourists’ behavior, demand forecasting, and satisfaction modeling. Li and Law [19] highlight the potential of big data in revolutionizing tourism marketing by offering a more granular understanding of consumer preferences and behaviors. Mariani [20] provide a thorough overview of the challenges and opportunities associated with big data in tourism and hospitality, noting that despite its potential, methodological and ethical issues remain significant barriers to its widespread adoption.

The impact of user-generated content (UGC) on destination image formation has become a central topic in contemporary tourism research. Online reviews and social media content have become critical components of destination marketing and play an important role in shaping tourist perceptions, as demonstrated by Chemin et al. [21]. Machine learning techniques have been increasingly employed to predict the sentiment of online reviews, providing a novel framework for comparative sentiment analysis, as proposed by Budhi, Chiong [22]. Furthermore, network analysis has been applied to tourism big data to uncover key research trends and emerging thematic clusters, thereby mapping the evolving landscape of tourist preferences and behaviors, as demonstrated by Li et al. [23].

Big data technologies have demonstrated considerable potential to enhance both theoretical understanding and practical applications in tourism research, as highlighted by Li and Law [19]. Topic-modeling-based sentiment classification has been shown to improve the accuracy of satisfaction assessment in consumer-generated online reviews, as demonstrated by Ali, Omar [24]. Furthermore, large-scale data analytics combining sentiment analysis and Latent Dirichlet Allocation (LDA) can provide valuable insights into the factors influencing tourist satisfaction and destination competitiveness, as evidenced by the study of Harbin’s ice and snow tourism conducted by Jiang et al. [25].

Despite a growing body of literature on ice and snow tourism, current research remains fragmented and methodologically limited, particularly in leveraging diverse data modalities and real-time analysis. Existing studies often rely on single-source data such as surveys or static textual reviews, which fall short in capturing the dynamic, multi-dimensional nature of tourist satisfaction. In response to these gaps, this study proposes an integrated analytical framework that combines user-generated reviews and sentiment analysis techniques to more accurately and dynamically assess tourist satisfaction in ice and snow tourism contexts. By addressing both spatial and emotional dimensions, the study provides a more nuanced understanding of tourist behavior and offers practical insights for destination management and service optimization.

Existing studies on ice and snow tourism and tourist satisfaction can be broadly categorized into three strands. The first strand focuses on climate dependency and sustainability, emphasizing the impacts of snow reliability, climate change, and adaptive strategies in traditional winter tourism destinations such as ski resorts in Europe and North America. The second strand examines tourist satisfaction determinants using survey-based or interview-based methods, highlighting the roles of service quality, infrastructure, and destination image, but largely treating satisfaction as a static outcome measured ex post. The third strand applies big data and sentiment analysis to tourism research, demonstrating the value of user-generated content for identifying experiential themes and emotional tendencies, yet often relying on cross-sectional designs and descriptive correlations. Despite these advances, several limitations remain. First, prior studies rarely integrate thematic cognition and emotional valence within a unified analytical framework, leaving their interaction insufficiently explored. Second, most existing sentiment-based studies focus on static associations and do not model the temporal dynamics or causal relationships between emotions and satisfaction. Third, research on ice and snow tourism has predominantly examined mature Western markets, while rapidly developing, policy-driven contexts such as China remain underrepresented. Consequently, there is a lack of evidence on how emotional fluctuations shape satisfaction formation in large-scale, highly seasonal, and digitally mediated tourism environments. To address these gaps, the present study proposes an integrated framework combining topic modeling, domain-adapted sentiment analysis, and vector autoregression. By leveraging large-scale longitudinal review data, this study explicitly models the dynamic and asymmetric effects of tourist emotions on satisfaction, thereby extending existing literature from static evaluation to a process-oriented understanding of satisfaction formation in ice and snow tourism.

### 2.4. Research gaps and study contributions

Existing studies on ice and snow tourism and tourist satisfaction have generated important insights into climate dependency, destination image, service quality, and tourist emotional experiences. However, several important research gaps remain.

First, previous studies have predominantly relied on survey-based or interview-based approaches, which often treat tourist satisfaction as a static post-consumption outcome. Such approaches have limited ability to capture the dynamic and evolving nature of tourists’ emotional experiences in highly seasonal tourism contexts.

Second, although recent studies have introduced sentiment analysis and text mining techniques into tourism research, most existing analyses remain descriptive and cross-sectional in nature. Limited attention has been paid to the temporal dynamics and causal interrelationships between emotional fluctuations and tourist satisfaction.

Third, prior research has largely focused on mature winter tourism markets in Europe and North America, while rapidly developing and policy-driven ice and snow tourism systems such as China remain underexplored. Consequently, the emotional mechanisms underlying tourist satisfaction formation in emerging winter tourism markets are still insufficiently understood.

To address these gaps, the present study proposes an integrated analytical framework combining LDA topic modeling, domain-adapted sentiment analysis, and Vector Autoregression (VAR) modeling. By leveraging large-scale longitudinal user-generated content from China’s ice and snow tourism destinations, this study contributes to the literature in three ways. First, it advances tourist satisfaction research from static evaluation toward a dynamic process-oriented perspective. Second, it integrates thematic cognition and emotional responses within a unified computational framework. Third, it empirically reveals the asymmetric and temporal effects of emotional fluctuations on tourist satisfaction in rapidly developing ice and snow tourism contexts. Table 1 summarizes the representative literature, major methodologies, key findings, and remaining limitations in existing studies related to ice and snow tourism and tourist satisfaction.

**Table 1:** Summary of prior studies on ice and snow tourism and tourist satisfaction.

Study focus	Representative studies	Data source	Methodology	Key findings	Limitations
Climate change & winter tourism sustainability	Scott, D., Hall, C. M., & Gössling, S. (2019); Steiger, R. et al. (2019)	Climate data, destination statistics	Scenario analysis; econometric modeling	Snow reliability significantly affects tourism demand, season length, and destination viability	Limited attention to tourist emotions and satisfaction mechanisms
Tourist satisfaction in winter destinations	Falk, M. (2010); Hosany, S. et al. (2015)	Surveys; interviews	Regression; structural equation modeling	Service quality, infrastructure, and destination image significantly influence satisfaction and loyalty	Satisfaction treated as static outcome; small or cross-sectional samples
Destination image & experiential perception	Kim, H., & Richardson, S. L. (2003); Chen, C. F., & Tsai, D. (2007)	Surveys; perception scales	Factor analysis; SEM	Destination image and perceived experience shape satisfaction and revisit intention	Limited temporal analysis; focus on cognitive evaluation
Big data & sentiment analysis in tourism	Li, X., & Law, R. (2020); Mariani, M. et al. (2018)	Online reviews (UGC)	Text mining; LDA; sentiment analysis	Identifies experiential themes and emotional polarity from large-scale online data	Mostly descriptive; limited causal modeling
Ice and snow tourism in China	Sun, X., Chi, C. G., & Xu, H. (2019); Jiang, Y. (2022)	Case studies; survey data	Qualitative analysis; regression	Rapid policy-driven growth; infrastructure pressure and seasonal concentration	Insufficient integration of emotional dynamics and temporal causality
This study	—	Longitudinal UGC (2018–2025)	LDA + sentiment analysis + VAR	Reveals dynamic and asymmetric emotional effects on satisfaction	Focused on China; platform-specific dataset

## 3. Materials and methods

This section introduces the study area, data sources, analytical framework, and methodological procedures employed in this research. It first describes the selected ice and snow tourism destinations and the collection and preprocessing of online review data. The section then presents the integrated analytical framework based on LDA topic modeling, sentiment analysis, and VAR modeling, followed by detailed explanations of the corresponding analytical methods.

### 3.1. Research area overview

China’s integration of ice and snow tourism into its national development strategy, accelerated by the 2022 Beijing Winter Olympics and the “300 million participants in ice and snow sports” initiative, has transformed the sector into a key driver of regional economic revitalization, particularly in northern and western regions, as highlighted by Tang et al [26].To analyze tourist satisfaction dynamics across diverse contexts, this study examines fifteen representative destinations across China. Deliberately selected for spatial representativeness and thematic diversity—encompassing urban attractions, ecological snowfields, alpine resorts, and rural villages—these sites include key locations in the Northeast (e.g., Harbin, Changchun), Northwest (Xinjiang), and North-Central regions (e.g., Hebei, Beijing). Their prominence on major travel platforms ensures high tourist traffic and robust data. This selection enables the investigation of spatial heterogeneity in satisfaction determinants, providing a nationally relevant framework for advancing seasonal tourism theory and practice in emerging markets.

### 3.2. Data sources and processing

The empirical foundation of this study is a large-scale dataset of user-generated content (UGC) collected from Ctrip (https://www.ctrip.com), one of the most influential online travel service and booking platforms in China. Following the data collection approach adopted by Xia et al. [27], Ctrip reviews were selected because they provide rich, large-scale, and experience-oriented textual information that is well suited for tourism satisfaction analysis. Fifteen iconic ice-and-snow tourism destinations across China were selected for analysis based on their established prominence in national tourism statistics, high digital visibility on travel platforms, and their geographical and experiential representativeness of the winter tourism sector (e.g., Harbin Ice and Snow World exemplifying large-scale ice architecture; Yabuli Ski Resort representing major ski destinations). Table 2 presents the full list of these destinations along with their respective review counts and proportional distributions, demonstrating the substantial coverage of China’s major ice and snow tourism markets.

**Table 2:** Distribution of online tourist reviews across selected ice and snow tourism destinations in China.

Region	Scenic Spot Name	Number of Reviews	Regional Total	Regional Proportion (%)
Heilongjiang	Harbin Ice and Snow World	5958	26362	37.53
	Saint Sophia Cathedral	5140		
	Harbin Polarland	6000		
	Volga Manor	4714		
	Arctic Village	2360		
	China Snow Town	2190		
Jilin	Changchun Ice and Snow New World	2350	6648	9.47
	Changbai Mountain	4298		
Xinjiang	Heavenly Lake of Tianshan	5998	23994	34.15
	Kanas Lake	6000		
	Sayram Lake	5996		
	Hemu Scenic Area	6000		
Beijing	Jundu Ski Resort	1518	1518	2.16
Hebei	Taiwu Ski Town	3066	6746	9.6
	Nanshan Ski Resort	3680		

We collected 32,634 Ctrip reviews (2018–2025) using a Python crawler. After deduplication, length filtering, and text normalization (removing non-Chinese elements, HTML, and emojis), domain-specific tokenization and stop-word removal were applied. A custom lexicon standardized tourism terminology (e.g., unifying festival/service synonyms). The final cleaned corpus of 32,516 reviews—annotated with ratings, timestamps, and destinations—was prepared for downstream analysis.

### 3.3. Research framework based on the CRISP-DM process

To improve methodological transparency and reproducibility, the analytical workflow of this study was conceptually aligned with the Cross-Industry Standard Process for Data Mining (CRISP-DM), a widely recognized framework for structuring data-driven research processes. The CRISP-DM methodology organizes analytical procedures into interconnected phases, including business understanding, data understanding, data preparation, modeling, evaluation, and deployment. Following this logic, the present study integrates text mining, sentiment analysis, and time-series modeling into a unified analytical framework for investigating tourist satisfaction dynamics in ice and snow tourism contexts.

**Fig 1:**
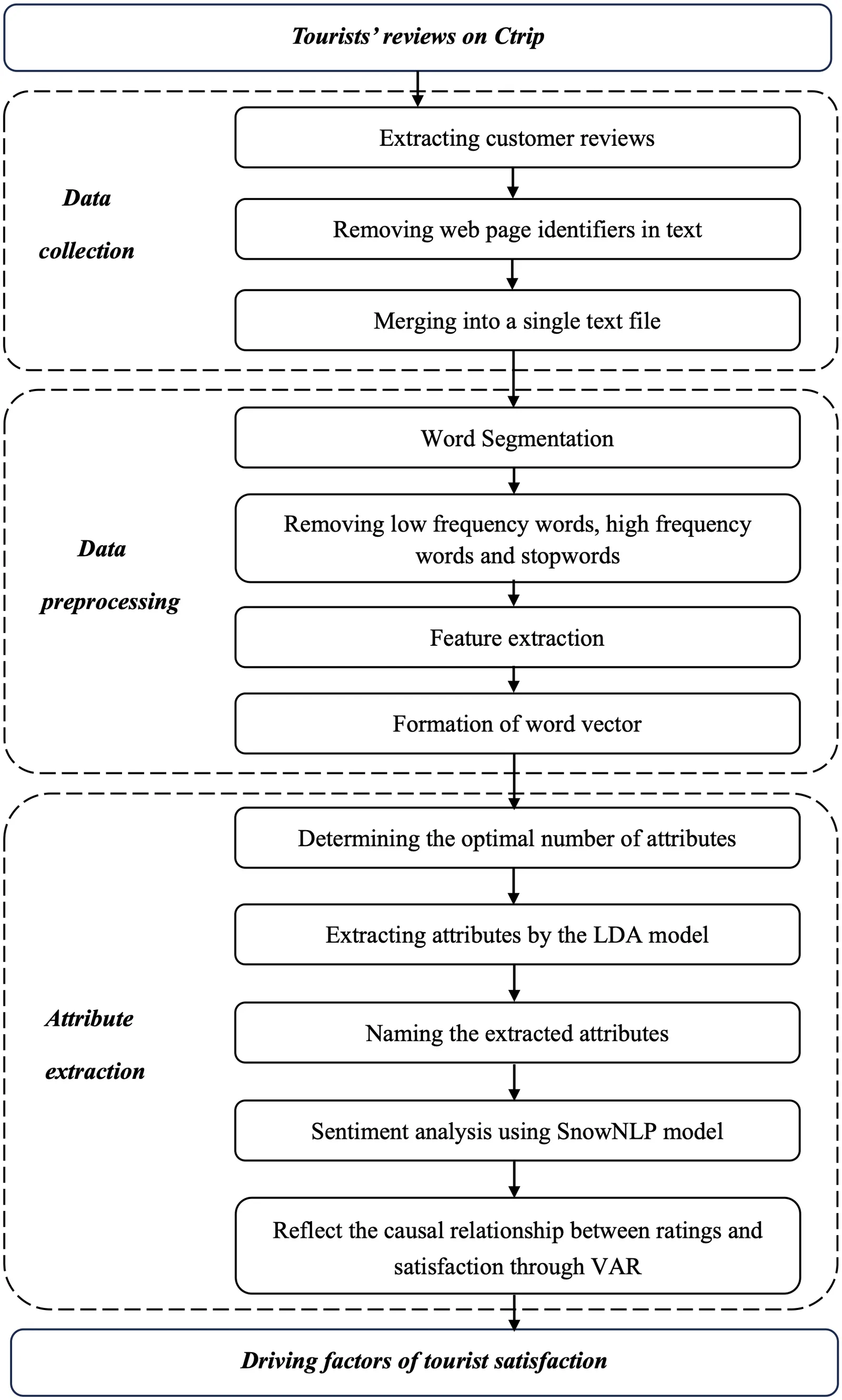
Research Framework Flowchart.

Fig 1 illustrates the overall research framework. The analytical workflow operationalizes the major phases of the CRISP-DM process through three interconnected stages: data collection and preparation, computational modeling, and evaluation and interpretation.

In the data collection and preparation stage, large-scale user-generated reviews were collected from the Ctrip platform and subjected to systematic preprocessing procedures, including duplicate removal, invalid text filtering, text normalization, tokenization, and stop-word removal. After data cleaning, 32,516 valid reviews from 2018 to 2025 were retained for subsequent analysis. The broad temporal coverage enabled the study to capture seasonal fluctuations and policy-related dynamics in tourists’ experiential evaluations.

In the computational modeling stage, the cleaned textual corpus underwent an integrated analysis combining LDA topic modeling and SnowNLP-based sentiment analysis. LDA was employed to identify latent experiential themes embedded in tourists’ online reviews, while sentiment analysis was used to quantify emotional valence associated with different tourism experiences. Through iterative coherence evaluation and semantic interpretation, ten dominant experiential dimensions were extracted, including natural ecology, polar wildlife, rural village landscapes, accommodation and transportation, mountain snow scenery, skiing facilities and equipment, service quality, ice and snow entertainment, snow-themed architecture, and fairy-tale atmosphere. Simultaneously, a domain-adapted SnowNLP model trained on manually labeled tourism reviews generated sentiment scores ranging from 0 (highly negative) to 1 (highly positive), allowing emotional characteristics to be associated with thematic dimensions.

In the evaluation and interpretation stage, the study further extended from descriptive text mining to dynamic causal analysis by incorporating topic-level sentiment indicators into a Vector Autoregression (VAR) framework. Monthly time-series variables were constructed to capture temporal fluctuations in tourist satisfaction dimensions. Granger causality tests and impulse response analyses (IRFs) were subsequently employed to examine the dynamic interrelationships and temporal effects among emotional experiences and satisfaction drivers. This integrated framework enables systematic interpretation of tourist satisfaction formation in China’s ice and snow tourism context and provides data-driven insights for destination management and tourism service optimization.

### 3.4. Research methods

#### 3.4.1. LDA model.

A Bayesian generative model was employed to identify latent topics within large document collections. This approach represents documents as mixtures of topics and has been widely adopted in topic modeling research, as originally proposed by Blei et al. [28]. It represents each document as a mixture of topics, while each topic is characterized by a probability distribution over words, as further discussed by Chang et al. [29]. In the present study, this flexible and noise-tolerant approach was applied to online reviews of ice and snow tourism destinations in Northeast China, following the application framework demonstrated by Marrese-Taylor et al. [30]. The analysis generated both document-topic and topic-term distribution matrices, providing the basis for subsequent thematic interpretation. Topic coherence optimization determined the optimal number of topics and their weights, enabling macro-level analysis of tourism characteristics to support subsequent sentiment analysis. As shown in Table 3, the coherence score reached its maximum at ten topics, which was therefore selected for subsequent analysis. Specifically, the Cv coherence metric was adopted because it has been widely recognized as an effective indicator for evaluating semantic interpretability in short tourism-related texts. Compared with perplexity-based evaluation, coherence scores better reflect human understanding of topic quality. The number of topics was determined through an iterative comparison of alternative models, followed by semantic validation to ensure conceptual clarity and practical interpretability.

**Table 3:** Topic coherence scores for candidate LDA models.

Number of Topics	Coherence Score
6	0.41
8	0.47
10	0.53
12	0.51
14	0.49

*Notes: Topic coherence was evaluated using the Cv coherence metric implemented in the Gensim library, which combines normalized pointwise mutual information (NPMI) with cosine similarity to assess semantic interpretability. Candidate topic numbers ranging from 6 to 14 were iteratively tested under the same hyperparameter settings. The model with 10 topics achieved the highest coherence score (0.53), indicating the best balance between semantic consistency and topic distinctiveness. In addition to the quantitative coherence evaluation, manual inspection by two tourism research scholars was conducted to verify the interpretability and thematic relevance of the extracted topics. Therefore, the 10-topic solution was selected for subsequent analysis.*

#### 3.4.2. SnowNLP sentiment analysis.

Sentiment analysis was performed using SnowNLP, a Python-based natural language processing library that has been widely applied in Chinese text sentiment classification, as reported by Zhang et al. [31]. Continuous sentiment scores ranging from 0 (highly negative) to 1 (highly positive) were generated, and the model was further optimized through domain-specific training to improve classification accuracy, following the approach proposed by Xu and Li [32].To optimize tourism context performance, the model was trained on 30,135 manually labeled Ctrip comments (26,082 positive; 4,053 negative) from comparable ice-snow destinations. The customized model subsequently computed: (1) raw comment-level sentiment scores, and (2) aggregated sentiment scores for each LDA-derived topic. These topic-level measurements provided foundational data for competitiveness analysis by integrating thematic content with emotional evaluations.

#### 3.4.3. VAR.

Vector Autoregression (VAR) is utilized to assess the temporal dynamics and interrelationships among sentiment-enhanced thematic variables. This method is particularly appropriate in the present research context because it treats all variables as endogenous and models their dynamic interactions over time without imposing strong a priori assumptions regarding causal directionality, consistent with the VAR framework developed by Sims [33]. Specifically, we specify a VAR(p) model of the form


$$\begin{array}{c} y_{t}=c+A_{\text{1}}y_{t-\text{1}}+\;\cdots +A_{p}y_{t-p}+\;e_{t} \end{array}$$ (1)

Where $y_{t}={\left(S_{t}\;,\;X_{\text{1},t}^{+}\;,\;X_{\text{1},t}^{\text{0}}\;,\;X_{\text{1},t}^{-}\;,\cdots ,X_{\text{1}\text{0},t}^{+},X_{\text{1}\text{0},t}^{\text{0}}\;,X_{\text{1}\text{0},t}^{-}\right)}^{⊺}\;$ is the k-dimensional vector ofendogenous variables in month t (k = 1 + 3 × 10 = 31); St denotes the overall satisfaction score in month t; $X_{i,t}^{+}\;,X_{i,t}^{\text{0}}\;,and\;X_{i,t}^{-}$ represent the positive,neutral,and negative sentiment scores, respectively, for theme i in month t, all derived from text-based sentiment analysis of online reviews; c is a k × 1 intercept vecto; Aj (j = 1,⋯,p) are k × k coefficient matrices capturing own-lag and cross-lag effects.

This setup treats all sentiment and satisfaction variables as jointly endogenous, allowing each series’ own lags and other series’ lags to explain its dynamics. Granger causality tests, originally proposed by Granger [34], were subsequently employed to determine the existence and direction of predictive relationships between variables. Generalized impulse response functions (IRFs), following the approach of Pesaran and Shin [35], were then used to visualize the effects of shocks in one variable (e.g., a decline in facility satisfaction) on other dimensions of tourist experience (e.g., service-quality perception) over a specified time horizon.The combination of these three methods provides a robust, interpretable, and policy-relevant approach for analyzing the complexity of tourist satisfaction in multi-dimensional and seasonally sensitive tourism contexts.

## 4. Results and discussion

This section presents the empirical findings derived from the integrated analytical framework. It first identifies the core experiential themes of ice and snow tourism destinations based on LDA topic modeling. Subsequently, seasonal sentiment dynamics are examined through sentiment analysis, followed by the analysis of temporal causal relationships among tourist satisfaction dimensions using the VAR model. The section concludes with a discussion of the theoretical and managerial implications of the findings.

### 4.1. The core experiential topics of ice and snow tourism

Latent Dirichlet Allocation (LDA) analysis of 32,516 online reviews identified ten core experiential dimensions characterizing tourists’ experiences in China’s ice and snow tourism destinations, including travel experience, ice and snow sports, architectural style, service facilities, natural landscape, interactive tours, snow landscape, outdoor scenery, accommodation and transportation, and parent-child entertainment.

**Fig 2:**
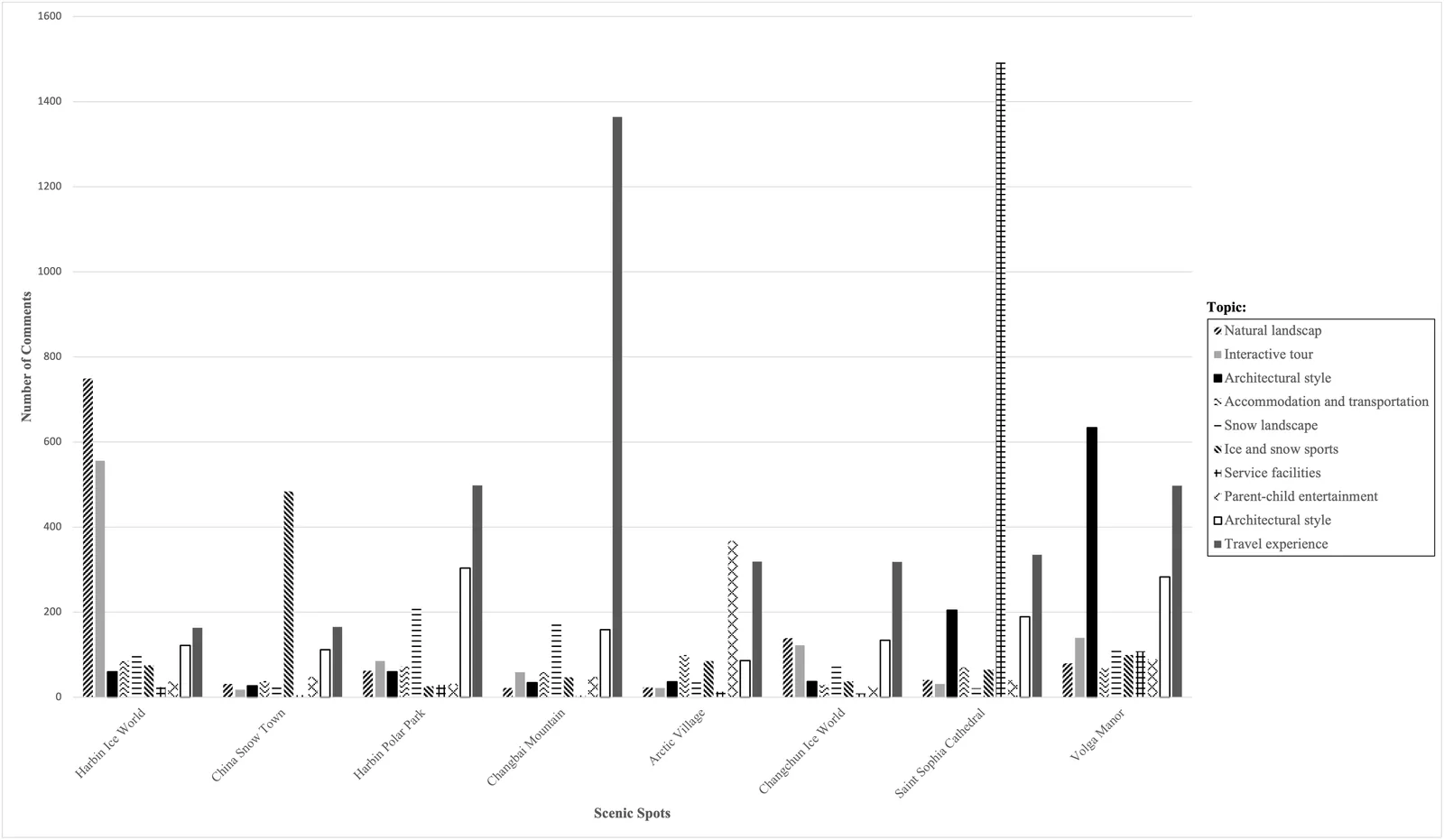
Thematic review distributions for representative ice and snow tourism destinations selected from the full sample of fifteen study sites.

Fig 2 presents the thematic review distributions for several representative ice and snow tourism destinations selected from the study sample. Clear differences in thematic emphasis can be observed across destinations, reflecting their unique tourism resources and market positioning. Harbin Polar Park received a particularly large number of reviews related to architectural style and interactive tourism experiences, indicating the importance of immersive attractions and visitor engagement activities. Saint Sophia Cathedral was strongly associated with architectural style and cultural landscape themes, highlighting its role as a landmark heritage attraction. In contrast, destinations characterized by natural resources, such as Changbai Mountain and Volga Manor, attracted a greater proportion of reviews related to snow landscapes, outdoor scenery, and travel experiences. China Snow Town exhibited a strong concentration of reviews associated with accommodation and transportation as well as travel experience, suggesting that infrastructure and visitor logistics play a significant role in shaping tourists’ evaluations.

Overall, the thematic distributions reveal substantial heterogeneity in tourist experiences across ice and snow tourism destinations. While some destinations are primarily valued for their natural and scenic resources, others derive their attractiveness from cultural heritage, recreational activities, interactive experiences, or family-oriented tourism products. These findings highlight the multidimensional nature of tourist satisfaction in China’s rapidly developing ice and snow tourism market.

Due to space limitations and readability considerations, only representative destinations are visualized in Fig 2, while the complete thematic distributions for all study destinations are provided in the Supplementary Material.

Crucially, the co-occurrence of activity-based (e.g., entertainment), environmental (scenery/ecology), cultural (village), and operational themes (transport/accommodation) in LDA results demonstrates that tourist satisfaction is driven by dynamic interactions among multidimensional factors—challenging traditional single-factor models. These findings highlight the need for holistic management strategies that integrate experience design and infrastructure development, particularly in addressing transportation and service bottlenecks during peak tourism seasons. Such improvements are essential for maintaining destination competitiveness and enhancing overall visitor satisfaction. Furthermore, the observed thematic disparities suggest that destination managers should adopt tailored regional development strategies that capitalize on unique local resources and experiential attributes, consistent with the experience economy framework proposed by Sun et al. [36]. Topic coherence analysis identified ten interpretable topics from the corpus of online reviews. Table 4 presents the LDA-derived topic categories and their representative high-probability keywords. The identified themes encompass both destination attributes and experiential dimensions, including travel experience, ice and snow sports, architectural style, service facilities, natural landscape, interactive tour, snow landscape, accommodation and transportation, and parent-child entertainment. Topic labels were assigned based on semantic interpretation of the highest-weighted keywords and validated through expert review.

**Table 4:** The results of topic model and keywords.

Topic	Topic Naming	The weight of top keywords
T1	Natural landscape	snow (0.084), forest (0.073), lake (0.069), ecology (0.065), environment (0.058), nature (0.054)
T2	Interactive tour	polar bear (0.091), penguin (0.087), seal (0.081), exhibit (0.074), children (0.062), performance (0.059)
T3	Architectural style	village (0.088), local (0.079), folk culture (0.072), street (0.066), architecture (0.061), atmosphere (0.057)
T4	Accommodation and transportation	hotel (0.089), transportation (0.081), shuttle bus (0.076), queue (0.070), check-in (0.064), traffic (0.060)
T5	Snow landscape	mountain (0.092), skiing slope (0.083), cable car (0.077), altitude (0.071), view (0.066), scenery (0.061)
T6	Ice and snow sports	ski (0.094), equipment (0.085), rental (0.078), boots (0.071), safety (0.067), beginner (0.062)
T7	Service facilities	service (0.090), staff (0.082), attitude (0.076), efficiency (0.070), management (0.065), experience (0.060)
T8	Parent-child entertainment	ice slide (0.093), snow sculpture (0.085), entertainment (0.079), festival (0.073), activity (0.068), fun (0.063)
T9	Architectural style	architecture (0.088), church (0.080), building (0.074), snow view (0.068), photography (0.063), landmark (0.058)
T10	Travel experience	fairy tale (0.091), magical (0.083), night view (0.076), lighting (0.070), atmosphere (0.065), romantic (0.061)

### 4.2. Seasonal sentiment analysis

**Fig 3:**
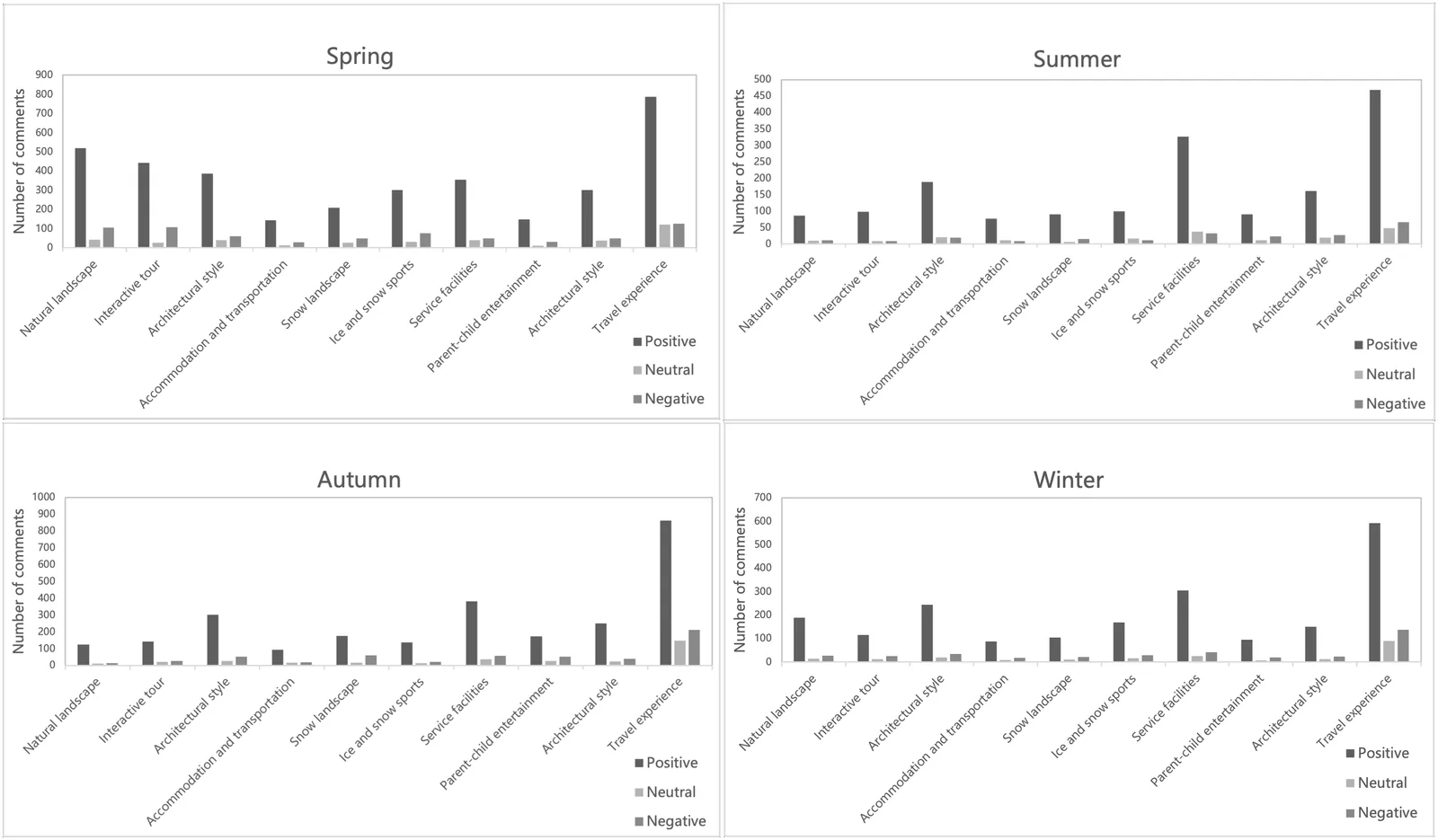
Seasonal sentiment distrbution by topic.

Sentiment analysis of 32,516 validated reviews revealed a robust positive skew across China’s ice-snow tourism sector (68.4% positive, 24.1% negative, and 7.5% neutral), indicating generally favorable tourism experiences. This distribution suggests that tourist satisfaction is closely associated with the degree of consistency between expected and actual experiences, a relationship that can be explained by the expectation-disconfirmation framework proposed by Oh et al. [37]. Significant seasonal heterogeneity was observed across experiential dimensions, demonstrating that satisfaction drivers vary substantially over time and that infrastructure and service systems play different roles across seasons, as illustrated in Fig 3.

*Spring:* Spring was characterized by strong positive sentiment toward Travel Experience, Natural Landscape, Interactive Tour, and Architectural Style. These results suggest that tourists highly valued the combination of natural scenery and cultural attractions during the shoulder season. However, comparatively higher negative sentiment was observed for Ice and Snow Sports and Service Facilities, indicating that some tourism facilities and winter-related recreational services were unable to fully satisfy visitor expectations after the peak winter season. This pattern is consistent with expectation-disconfirmation theory, which suggests that dissatisfaction may arise when actual service performance falls below anticipated standards, as explained by Oliver [38].

*Summer:* Summer exhibited a mixed sentiment pattern. Positive evaluations remained high for Travel Experience, Service Facilities, and Architectural Style, reflecting the benefits of lower visitor pressure and improved service accessibility during the off-season. In contrast, Natural Landscape and Interactive Tour attracted relatively higher levels of negative sentiment compared with other topics. The reduced availability of snow-related tourism resources and the discrepancy between winter-oriented destination images and actual summer conditions may contribute to this dissatisfaction. Such findings highlight the importance of destination image management and expectation alignment, as discussed by De Bloom et al. [39].

*Autumn:* Autumn: Tourists expressed particularly positive sentiment toward Natural Ecology, Village Landscape, and Snow Mountain Scenery, suggesting strong appreciation for transitional seasonal landscapes. Nevertheless, Accommodation and Transportation and Service Evaluation attracted comparatively higher negative sentiment. Resource constraints and preparations for the upcoming winter season may reduce service efficiency, reinforcing the importance of transportation accessibility and service quality as key determinants of tourist satisfaction, as emphasized by Buttle and Francis [40].

*Winter:* Winter displayed the strongest emotional polarization among all seasons. Travel Experience, Accommodation and Transportation, and Service Facilities generated exceptionally high levels of positive sentiment, reflecting tourists’ immersion in winter recreational activities and destination experiences. At the same time, these dimensions also received relatively high levels of negative sentiment, indicating that crowding, capacity limitations, and operational pressure during the peak tourism season may adversely affect visitor experiences. This coexistence of intense positive and negative emotions highlights the critical role of service quality management in maintaining tourist satisfaction during periods of peak demand, as discussed by De Bloom et al. [39].

Destination-level analysis further contextualized these seasonal dynamics. Harbin Ice and Snow World generated particularly strong positive sentiment regarding Travel Experience and Ice and Snow Sports, reflecting its distinctive winter recreation attractions. Harbin Polar Park was characterized by high levels of positive sentiment associated with Interactive Tour experiences, whereas destinations such as Changbai Mountain and Saint Sophia Cathedral received consistently favorable evaluations for Natural Landscape and Architectural Style, respectively. Cross-thematic analysis further suggested that positive evaluations of Travel Experience were frequently accompanied by positive perceptions of Natural Landscape and Service Facilities, indicating that immersive tourism experiences may enhance tourists’ overall evaluations of destination attractiveness.

### 4.3. Empirical results of VAR

Vector Autoregression (VAR) modeling reveals dynamic interdependencies among tourism satisfaction factors, with satisfaction measured using ratings. Granger causality tests establish that satisfaction with “Ice and Snow Sports” is a significant Granger cause for satisfaction with “Natural Landscape” (p < 0.01), “Snow Landscape” (p < 0.05), and “Service Facilities” (p < 0.01), indicating winter activities systematically enhance environmental and service evaluations. Crucially, bidirectional causality links satisfaction with “Natural Landscape” and “Ice and Snow Sports” (p < 0.01), empirically validating the activity-environment reciprocity mechanism where recreational immersion and environmental appreciation reinforce each other. Meanwhile, satisfaction with “Accommodation and Transportation” functions as both driver and outcome—significantly Granger-causing satisfaction with “Service Facilities” (p < 0.01) and “Travel Experience” (p < 0.05), while being significantly Granger-caused by satisfaction with “Ice and Snow Sports” (p < 0.05) and “Snow Landscape” (p < 0.01), highlighting infrastructure’s critical role in the satisfaction valuation process.

**Fig 4:**
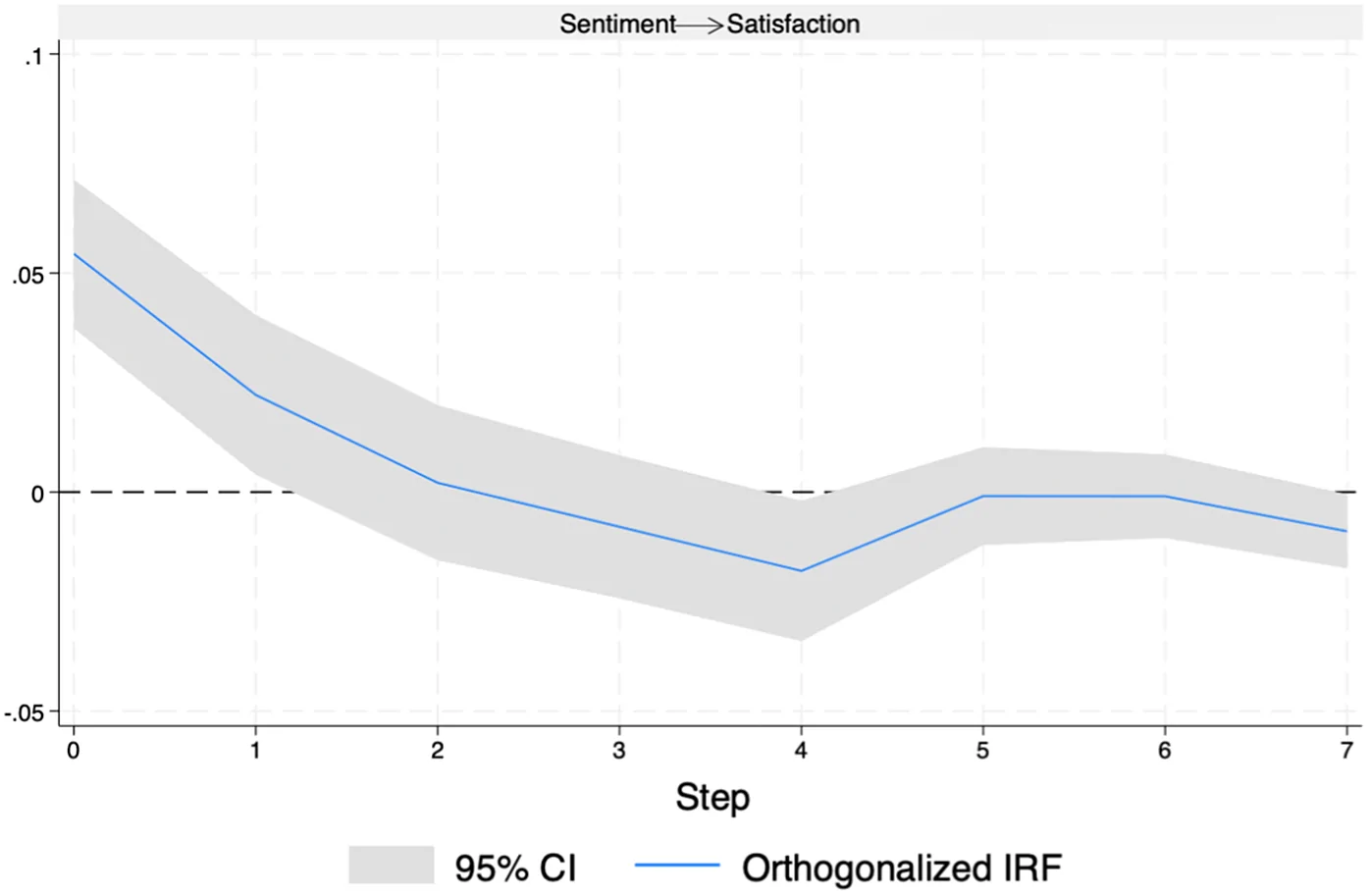
Impulse Response Analysis.

Impulse response analysis demonstrates that a one-standard-deviation shock to aggregate tourist sentiment generates a sustained satisfaction increase, stabilizing at +0.82 rating points by period 5 (p < 0.001), as visualized in Fig 4. This confirms sentiment as a temporally antecedent driver and establishes the sentiment-rating inertia mechanism (IRF Δ = +0.82, p < 0.001). The persistent positive trajectory indicates that initial sentiment improvements create self-reinforcing satisfaction loops, with effects remaining significant throughout the observed horizon.

These findings establish satisfaction as a multidimensional construct driven by recursive sentiment-rating linkages (inertia). The activity-environment synergy (where snow sports catalyze spillovers) and infrastructure’s critical mediation reveal inherent temporal sensitivity, with rating stabilization demonstrating experiential reevaluation inertia. Holistic management must therefore synchronize activity-environment integration with preemptive mitigation of logistical bottlenecks during peak demand to capitalize on cross-dimensional synergies. Regional LDA disparities (e.g., Harbin’s entertainment-architecture vs. Sayram Lake’s scenery-village focus) further modulate these pathways, reframing satisfaction as a spatiotemporally contextualized outcome of recursive environment-infrastructure-sentiment interactions.

## 5. Conclusions and implication

This study empirically deciphers the emotional dynamics, experiential concerns, and satisfaction pathways in China’s ice and snow tourism through the integrated analysis of 32,516 online reviews. By combining topic modeling, sentiment analysis, and vector autoregression techniques, the research provides a comprehensive and data-driven understanding of how satisfaction is formed in highly seasonal and emotionally intensive tourism environments. The findings reveal that tourists exhibit significantly polarized emotional experiences. On the one hand, strong positive sentiment is associated with immersive ice and snow entertainment activities, scenic landscapes, and fairy-tale-like atmospheres, with 68.4% of reviews expressing positive emotions. On the other hand, negative sentiment emerges prominently in relation to service infrastructure particularly accommodation and transportation during peak demand periods. This pattern reflects the high emotional volatility characteristic of weather-dependent and capacity-constrained tourism settings. The analysis further identifies infrastructure-related concerns as critical determinants of dissatisfaction. Issues related to accommodation, transportation, and perceived pricing fairness become especially salient during transitional and peak seasons, accounting for a substantial proportion of negative sentiment, with autumn alone contributing 12.19% of total negative expressions. These findings suggest that tourists’ evaluations extend beyond natural snow conditions to include operational reliability and fairness in service delivery. More importantly, the empirical results demonstrate a clear and statistically robust pathway from emotional experience to satisfaction. Granger causality tests indicate a unidirectional relationship in which sentiment significantly influences satisfaction ratings (χ²(4) = 21.32, p < 0.001), while the reverse effect is not statistically significant (χ²(4) = 6.46, p = 0.168). The vector autoregression model further confirms that a one-standard-deviation improvement in sentiment increases satisfaction ratings by 0.82 units (p < 0.001), with the effect stabilizing after approximately five time periods. These results indicate that emotional responses—particularly negative ones serve as primary antecedents in the satisfaction formation process rather than as outcomes of evaluative judgments. Collectively, the study demonstrates that tourist satisfaction in ice and snow destinations is shaped by dynamic and asymmetric emotional fluctuations embedded within seasonal and operational contexts. By integrating thematic cognition, sentiment polarity, and temporal causality modeling, this research provides an empirically grounded explanation of how experiential perceptions translate into satisfaction outcomes in complex winter tourism systems.

*Theoretical implication:* This study contributes to the tourism satisfaction literature in three important theoretical respects. First, the findings advance understanding of the affect-driven satisfaction formation mechanism in ice and snow tourism. Rather than viewing satisfaction solely as an evaluation of destination attributes, the results demonstrate that emotional experiences generated through interactions with natural landscapes, recreational activities, and tourism services constitute the primary foundation of satisfaction formation. The findings suggest that tourist satisfaction is fundamentally affective in nature, with emotional responses serving as a key mediating mechanism through which destination experiences are translated into overall evaluations. Second, this study extends existing satisfaction theories by conceptualizing tourist satisfaction as a dynamic and path-dependent process rather than a static post-consumption outcome. The temporal relationships identified among different experiential dimensions indicate that satisfaction develops through cumulative interactions between emotions, experiences, and service encounters over time. This finding challenges traditional cross-sectional perspectives and highlights the importance of understanding satisfaction as an evolving process characterized by feedback effects and temporal dependencies. Third, the study provides empirical evidence for emotional asymmetry in winter tourism experiences. The results reveal that negative emotions associated with service failures, crowding, and infrastructure constraints exert a disproportionately stronger influence on overall satisfaction than positive emotions generated by recreational experiences. This asymmetrical effect suggests that negative emotional experiences possess greater explanatory power in shaping tourist evaluations and behavioral responses. Consequently, emotional risk factors should occupy a more central position within theoretical models of tourism satisfaction and destination management. Taken together, these findings shift the focus of tourism satisfaction research from methodological measurement toward the underlying emotional and dynamic mechanisms that govern satisfaction formation, thereby enriching theoretical understanding of tourist behavior in winter tourism contexts.

*Practical implications.* From a managerial and policy perspective, the findings of this study have several important implications for destination management organizations (DMOs), policymakers, and tourism practitioners, particularly in cold-region and weather-sensitive tourism markets. First, the identification of sentiment as a primary driver of satisfaction indicates the need for developing real-time monitoring systems that can track emotional feedback across key service dimensions such as infrastructure, customer service, and recreational experiences. These systems can help detect dissatisfaction trends early and support proactive interventions, especially during high-volume periods when operational strain tends to peak. Second, the finding that negative sentiment is most frequently triggered by perceptions of pricing unfairness underscores the importance of implementing transparent, flexible, and value-sensitive pricing strategies. Price structures that reflect real-time demand and perceived service value can help manage tourist expectations and reduce emotional dissonance. Adopting dynamic pricing models based on sentiment analysis and demand elasticity could also enhance price acceptance and brand trust. Third, the sequential structure revealed by the VAR model suggests that investment in one aspect of the tourism experience—such as transportation facilities—can have delayed but significant effects on satisfaction in other areas, such as service evaluation and environmental perception. This provides empirical support for temporally coordinated planning strategies and resource allocation in tourism infrastructure development.

*Limitations and Future Research Directions.* Looking ahead, this study offers several avenues for future research and acknowledges certain limitations that merit further exploration. First, the present study relies exclusively on user-generated reviews collected from Ctrip, a major Chinese online travel platform primarily used by domestic tourists. As a result, the emotional expressions, consumption preferences, and satisfaction evaluations reflected in the dataset are likely shaped by specific cultural, linguistic, and platform-related characteristics associated with Chinese tourism contexts. Consequently, the findings should be interpreted with caution when extending them to international tourism markets or cross-cultural settings. Nevertheless, given that China currently represents one of the world’s fastest-growing ice and snow tourism markets under strong policy support and rapid digitalization, the Chinese context provides important empirical value for understanding the dynamics of emerging winter tourism systems. Future studies could improve the external validity and cross-cultural generalizability of the findings by incorporating multilingual and multi-platform datasets from international tourism platforms such as TripAdvisor, Booking.com, and Google Reviews. Second, although the SnowNLP tool demonstrates satisfactory performance for sentiment classification in Chinese, its dictionary-based structure may not fully capture the subtle emotional expressions and context-specific meanings in natural language. Future research could adopt transformer-based deep learning models, such as BERT or RoBERTa, to improve the accuracy and depth of sentiment analysis. Third, the current use of a vector autoregression (VAR) model assumes linear and stationary relationships, which may oversimplify the complex, time-varying dynamics of tourist behavior. More advanced modeling approaches, including Bayesian structural time series or nonlinear dynamic models, could offer a more flexible and realistic representation. In addition, the present study employs a standard VAR framework that primarily captures lagged temporal interactions among sentiment and satisfaction variables. Although this approach is effective for exploring dynamic associations, it does not explicitly identify contemporaneous structural relationships or exogenous shocks among variables. Future research could extend the current framework by adopting Structural Vector Autoregression (SVAR) models to better distinguish structural effects and enhance the robustness and interpretability of impulse response analysis. Finally, with the growing influence of climate variability, pandemics, and geopolitical disruptions on winter tourism, future studies should adopt longitudinal designs to examine how tourist emotions, satisfaction, and behavioral patterns evolve in response to these external shocks. Such research would provide valuable insights for building more resilient and adaptive tourism systems.

## References

[1] Jiang H. The impact of ice and snow sports on regional tourism economy and social development—A case study of Northeast China. Clausius Press. 2024.

[2] CGTN. China’s ice-snow economy boosts winter consumption. 2024.

[3] François H, Samacoïts R, Bird DN, Köberl J, Prettenthaler F, Morin S. Climate change exacerbates snow-water-energy challenges for European ski tourism. Nat Clim Chang. 2023;13(9):935–42. doi: 10.1038/s41558-023-01759-5

[4] Richins H. Winter tourism development and sustainability: regional development, ownership and infrastructure; environmental management and sustainability; historical and future perspectives, trends and implications, in Winter tourism: trends and challenges. CABI Wallingford UK. 2019. p. 291–304.

[5] Huang X, Chelliah S. Attributes influencing tourist satisfaction: Sentiment analysis and topic modeling of online reviews. Journal of China Tourism Research. 2024;:1–20.

[6] Guo Y, Barnes SJ, Jia Q. Mining meaning from online ratings and reviews: Tourist satisfaction analysis using latent dirichlet allocation. Tourism management. 2017;**59**:467–83.

[7] Scott D, Hall CM, Gössling S. Global tourism vulnerability to climate change. Ann Tourism Res. 2019;77:49–61. doi: 10.1016/j.annals.2019.05.007

[8] Steiger R, Scott D, Abegg B, Pons M, Aall C. A critical review of climate change risk for ski tourism. Current Issues in Tourism. 2017;22(11):1343–79. doi: 10.1080/13683500.2017.1410110

[9] Timoshenko DS. Sustainable tourism development in the russian arctic: challenges and prospects. IOP Conf Ser: Earth Environ Sci. 2020;539(1):012097. doi: 10.1088/1755-1315/539/1/012097

[10] Hao F. The landscape of customer engagement in hospitality and tourism: a systematic review. IJCHM. 2020;32(5):1837–60. doi: 10.1108/ijchm-09-2019-0765

[11] Richards G. Cultural tourism: A review of recent research and trends. Journal of Hospitality and Tourism Management. 2018;36:12–21. doi: 10.1016/j.jhtm.2018.03.005

[12] Dawson J, Scott D. Managing for climate change in the alpine ski sector. Tourism Management. 2013;35:244–54. doi: 10.1016/j.tourman.2012.07.009

[13] Liu S, Guo Q. Image perception of ice and snow tourism in China and the impact of the Winter Olympics. PLoS One. 2023;18(6):e0287530. doi: 10.1371/journal.pone.0287530 37352278 PMC10289359

[14] Falk M. A dynamic panel data analysis of snow depth and winter tourism. Tourism Manag. 2010;31(6):912–24. doi: 10.1016/j.tourman.2009.11.010

[15] Kim H, Stepchenkova S. Effect of tourist photographs on attitudes towards destination: Manifest and latent content. Tourism Management. 2015;49:29–41. doi: 10.1016/j.tourman.2015.02.004

[16] Hosany S, et al. Measuring tourists’ emotional experiences: further validation of the destination emotion scale. J Travel Res. 2015;54(4):482–95.

[17] Zhang B. The analysis of ecological security and tourist satisfaction of ice-and-snow tourism under deep learning and the Internet of Things. Sci Rep. 2024;14(1):10705. doi: 10.1038/s41598-024-61598-y 38730047 PMC11087544

[18] Puh K, Bagić Babac M. Predicting sentiment and rating of tourist reviews using machine learning. JHTI. 2022;6(3):1188–204. doi: 10.1108/jhti-02-2022-0078

[19] Li X, Law R. Network analysis of big data research in tourism. Tourism Management Perspectives. 2020;33:100608. doi: 10.1016/j.tmp.2019.100608

[20] Mariani M. Big Data and analytics in tourism and hospitality: a perspective article. TR. 2019;75(1):299–303. doi: 10.1108/tr-06-2019-0259

[21] Chemin M, Silva CP da, Vikou SV de P. User‑generated content (UGC) in tourist attractions and destinations: systematic literature review and perspectives for management. pasos. 2025;23(2):539–62. doi: 10.25145/j.pasos.2025.23.036

[22] Budhi GS, Chiong R, Pranata I, Hu Z. Using Machine Learning to Predict the Sentiment of Online Reviews: A New Framework for Comparative Analysis. Arch Computat Methods Eng. 2021;28(4):2543–66. doi: 10.1007/s11831-020-09464-8

[23] Li J, Xu L, Tang L, Wang S, Li L. Big data in tourism research: A literature review. Tourism Manag. 2018;68:301–23. doi: 10.1016/j.tourman.2018.03.009

[24] Ali T, Omar B, Soulaimane K. Analyzing tourism reviews using an LDA topic-based sentiment analysis approach. MethodsX. 2022;9:101894. doi: 10.1016/j.mex.2022.101894 36405368 PMC9672446

[25] Jiang B, Zhang C, Cui Y, Zhu J, Liu Z. Enhancement of Harbin ice and snow tourism destination competitiveness: A large-scale data study based on sentiment analysis and Latent Dirichlet Allocation. PLoS One. 2025;20(3):e0319435. doi: 10.1371/journal.pone.0319435 40117293 PMC11927900

[26] Chengcai T, Rui Z, Yuanyuan Y, Shiyi X, Xin W. High-Quality Development Paths of Ice-Snow Tourism in China from the Perspective of the Winter Olympics. J Resources Ecol. 2022;13(4). doi: 10.5814/j.issn.1674-764x.2022.04.002

[27] Xia H, et al. Smart recommendation for tourist hotels based on multidimensional information: a deep neural network model. J Tourism and Hospitality Manag. 2023.

[28] Blei DM, Ng AY, Jordan MI. Latent Dirichlet allocation. J Machine Learning Res. 2003;3(4–5):993–1022.

[29] Chang I-C, Yu T-K, Chang Y-J, Yu T-Y. Applying Text Mining, Clustering Analysis, and Latent Dirichlet Allocation Techniques for Topic Classification of Environmental Education Journals. Sustainability. 2021;13(19):10856. doi: 10.3390/su131910856

[30] Marrese-Taylor E, Velásquez JD, Bravo-Marquez F. A novel deterministic approach for aspect-based opinion mining in tourism products reviews. Expert Systems with Applications. 2014;41(17):7764–75. doi: 10.1016/j.eswa.2014.05.045

[31] Zhang L, Wang S, Liu B. Deep learning for sentiment analysis: A survey. Wiley Interdisciplinary Reviews: Data Mining and Knowledge Discovery. 2018;8(4):e1253.

[32] Xu X, Li Y. The antecedents of customer satisfaction and dissatisfaction toward various types of hotels: A text mining approach. International Journal of Hospitality Management. 2016;55:57–69. doi: 10.1016/j.ijhm.2016.03.003

[33] Sims CA. Macroeconomics and Reality. Econometrica. 1980;48(1):1–48.

[34] Granger CWJ. Investigating Causal Relations by Econometric Models and Cross-spectral Methods. Econometrica. 1969;37(3):424. doi: 10.2307/1912791

[35] Pesaran HH, Shin Y. Generalized impulse response analysis in linear multivariate models. Economics Letters. 1998;58(1):17–29. doi: 10.1016/s0165-1765(97)00214-0

[36] Sun K, Tian X, Xia J, Ou M, Tang C. The Market Responses of Ice and Snow Destinations to Southerners’ Tourism Willingness: A Case Study from China. Sustainability. 2023;15(18):13759. doi: 10.3390/su151813759

[37] Oh H, Fiore AM, Jeong M. Measuring experience economy concepts: tourism applications. J Travel Res. 2023;62(7):1619. doi: 10.1177/00472875211012345

[38] Oliver RL. A Cognitive Model of the Antecedents and Consequences of Satisfaction Decisions. Journal of Marketing Research. 1980;17(4):460. doi: 10.2307/3150499

[39] de Bloom J, Geurts SAE, Taris TW, Sonnentag S, de Weerth C, Kompier MAJ. Effects of vacation from work on health and well-being: Lots of fun, quickly gone. Work & Stress. 2010;24(2):196–216. doi: 10.1080/02678373.2010.493385

[40] Buttle I, Francis K. SERVQUAL: review, critique, research agenda. European J Marketing. 1996;30(1):8–32.

